# Podargiform Affinities of the Enigmatic *Fluvioviridavis platyrhamphus* and the Early Diversification of Strisores (“Caprimulgiformes” + Apodiformes)

**DOI:** 10.1371/journal.pone.0026350

**Published:** 2011-11-30

**Authors:** Sterling J. Nesbitt, Daniel T. Ksepka, Julia A. Clarke

**Affiliations:** 1 Department of Geological Sciences, Jackson School of Geosciences, The University of Texas at Austin, Austin, Texas, United States of America; 2 Department of Marine, Earth, and Atmospheric Sciences, North Carolina State University, Raleigh, North Carolina, United States of America; 3 Department of Paleontology, North Carolina Museum of Natural Sciences, Raleigh, North Carolina, United States of America; University of Lethbridge, Canada

## Abstract

**Background:**

The early Eocene Green River Formation avifauna preserves exceptional exemplars of the earliest unambiguous stem representatives of many extant avian clades. We identify the basal-most member of Podargiformes (extant and fossil stem lineage frogmouths) based on a new specimen of *Fluvioviridavis platyrhamphus*, a unique neoavian bird from the Fossil Butte Member of the Green River Formation of Wyoming. Extant frogmouths (Podargidae) comprise approximately 13 nocturnal species with an exclusively Australasian distribution.

**Methodology/Principal Findings:**

The new specimen was included in a combined phylogenetic analysis of morphological (osteology and soft tissue) and molecular sequence (cytochrome b, c-myc exon 3, and RAG) data sampling species-level taxa from both extant and extinct members of Steatornithidae, Podargidae, Caprimulgidae, Nyctibiidae, Aegothelidae, and Apodiformes ( = Strisores). New data from *F. platyrhamphus* help resolve phylogenetic relationships within Strisores, supporting placement of *F. platyrhamphus* and the European fossil form *Masillapodargus longipes* as basal parts of Podargiformes and also supporting a sister taxon relationship between Podargiformes and Steatornithiformes (oilbirds) within Strisores. This relationship is recovered only when fossil taxa are included, reaffirming the potential impact of stem fossil taxa on inferences of phylogenetic relationships. The well-preserved mandible and palate of the new specimen demonstrate that many of the unique characteristics of the skull that characterize the crown frogmouth clade Podargidae arose early in the evolutionary history of the clade, over 50 million years ago. Comparisons with the new specimen also indicate that *Eurofluvioviridavis* and *Fluvioviridavis* are not closely related.

**Conclusions/Significance:**

Together with the European fossil frogmouth *Masillapodargus*, *Fluvioviridavis* shows that Podargiformes had a much wider geographic distribution in the past, whereas extant species are restricted to Australasia. The Eocene record of Strisores from the Green River Formation and Messel Formation indicates most major subclade divergences had already occurred by the early-middle Eocene.

## Introduction

Avian fossils from the Green River Formation provide one of the most complete windows into a Paleogene avifauna worldwide. Although some early accounts of Green River birds based on incomplete materials and non-cladistic methodologies resulted in vague or erroneous estimates of avifauna composition, today the affinities of many of these taxa have been resolved and most have been identified as stem members of various extant avian subclades. Yet, several enigmatic taxa have resisted placement within the context of avian phylogeny (e.g., *Foro panarium*). One of the most challenging taxa to place has been *Fluvioviridavis platyrhamphus* (literally “broad-billed Green River bird”). This wide-beaked, short-legged taxon has been interpreted variably as a possible relative of rollers [1], oilbirds [2] or a basal higher land bird of indeterminate affinities [3].

*Fluvioviridavis platyrhamphus* has until now been known from a single, partially articulated individual from the Eocene Green River Formation of Wyoming [3]. The holotype specimen (SMNK.PAL.2368a+b) consists of much of the skeleton including a skull preserved in dorsal view, both wings and hind limbs, the pelvic girdle, and much of the pectoral girdle (figure 1 of [3]). However, many of the elements have poorly preserved surfaces and details of the pectoral girdle, palate, and hind limbs are not visible. Mayr and Daniels [3] observed similarities to both Steatornithidae (oilbird) and the broad-billed roller *Eurystomus*, but did not assign the taxon to a specific clade. These authors further noted similarities among *Fluvioviridavis* and isolated specimens from Messel (e.g., SMF-ME 10783a+b) and the London Clay (privately held specimens), which they tentatively referred to *Fluvioviridavis* sp. In a subsequent paper, Mayr [4] coined Fluvioviridavidae to include *Fluvioviridavis platyrhamphus* and two specimens from the Messel Formation, the holotype specimen of the new taxon *Eurofluvioviridavis robustipes* and a referred skull. In that work, Mayr [4] combined scorings from *Fluvioviridavis* and *Eurofluvioviridavis* into a single supraspecific terminal (Fluvioviridavidae), which was included in a phylogenetic analysis sampling much of the diversity of Aves with higher taxon composite terminals. Fluvioviridavidae was recovered as the sister taxon to a large clade of “higher land birds” including Psittaciformes, Piciformes, Passeriformes, Cuculiformes, Leptosomidae, and the traditional contents of "Coraciiformes". Cypselomorphae (Caprimulgidae, Nyctibiidae, Aegothelidae, and Apodiformes) was recovered outside of this clade. Notably, Podargidae clustered within the large “higher land bird” clade, rather than with Steatornithidae or Cypselomorphae. Support values were weak, however, and when monophyly of Neoaves was constrained, most relevant branches collapsed. More recently, Mayr [5] tentatively proposed that Fluvioviridavidae may be closely related to Strisores based on overall similarity of the wide beak and the short legs, but also noted that the gross differences between *Eurofluvioviridavis* and *Fluvioviridavis* might preclude an assignment of *Eurofluvioviridavis* to Fluvioviridavidae.

**Figure 1:**
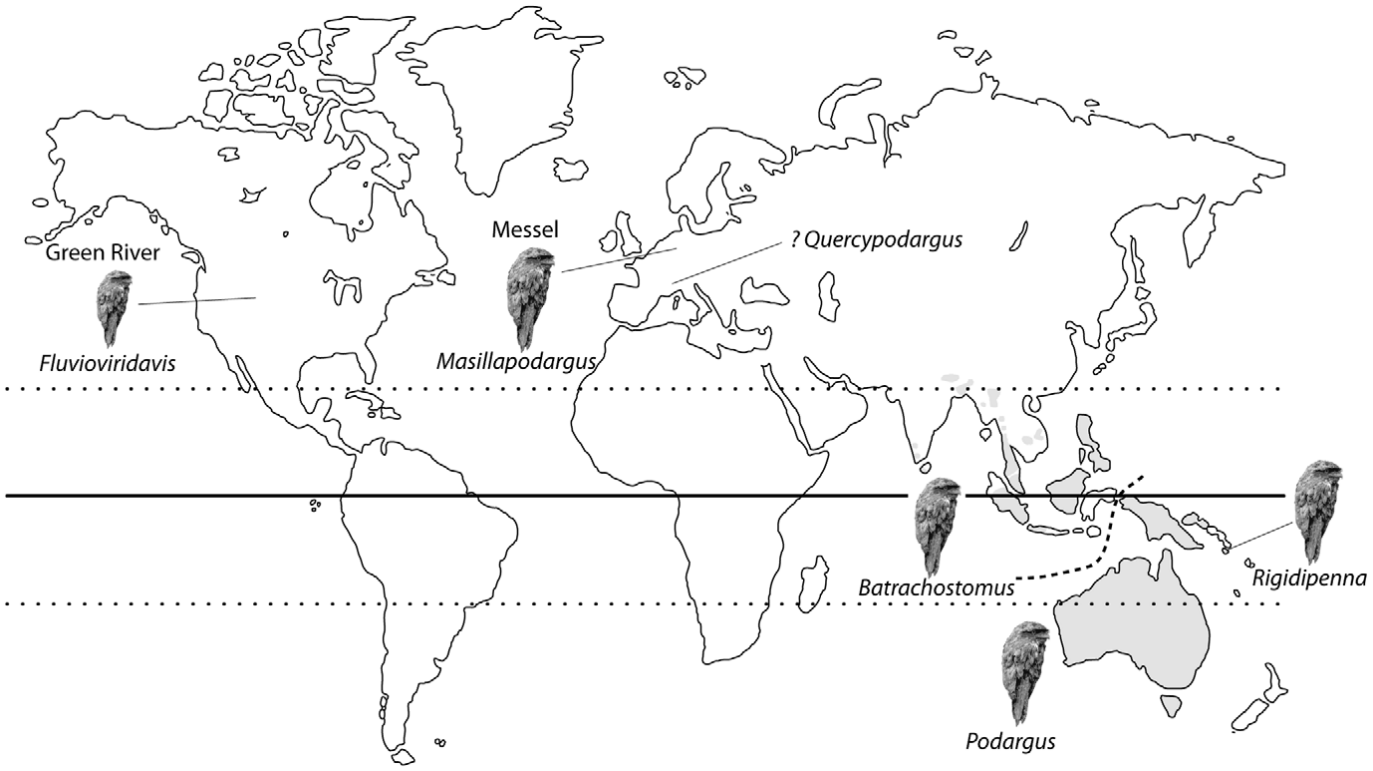
Map showing the distribution of extant Podargidae (light grey, after [**6**]) and localities yielding fossil Podargiformes (dark grey circles). The dashed line separates the ranges of extant *Batrachostomus* and *Podargus*.

Here, we describe a well preserved, articulated specimen assignable to *Fluvioviridavis platyrhamphus* from the Fossil Butte Member of the Green River Formation. Previously, this specimen was mentioned as likely referable to Fluvioviridavidae [5], but was not assigned to *Fluvioviridavis.* The new specimen preserves details of the palate, mandible, vertebrae, and hind limb not available in the holotype. The anatomical features of both specimens of *Fluvioviridavis* support a close relationship to Podargidae (frogmouths). We provide a new phylogenetic analysis of Strisores including extinct representatives.

### Living and Fossil Podargiformes

Podargidae are nocturnal arboreal birds characterized by their remarkable camouflaging plumage and eponymous wide, frog-like gape. The 13 extant species are distributed throughout continental Southeast Asia, Australia and numerous Australasian islands (Fig. 1), occurring primarily in forested environments, but also more open wooded savannas and scrublands [6]. Due to their secretive nature and nocturnal activity patterns, the habits of these birds remain poorly known, but most species appear to subsist primarily on insects captured during sallies from perches. The larger species of *Podargus* also prey on frogs, lizards and rodents [6]. Extant species diversity is currently divided into three genera. The longer-billed, large frogmouths of *Podargus* have long been separated from *Batrachostomus*, a taxon that spans a wide size range including the smallest living frogmouths. Most recently, the genus *Rigidipenna* was erected for the stiff-feathered Solomon Islands species *Rigidipenna inexpectata*, which can be separated from other living frogmouth species based on osteological and plumage traits as well as sequence divergence [7].

Fossil representatives of the frogmouth lineage are exceedingly rare (Fig. 1). Two European fossil species of stem Podargiformes have been previously recognized. Of these, the middle Eocene Messel frogmouth *Masillapodargus longipes* [8], [9] is the best known. *Masillapodargus longipes* exhibits the derived wide, flattened bill of living frogmouths, though plesiomorphic features from other areas of the skeleton indicate a stem position for this taxon [8]. The late Eocene *Quercypodargus olsoni* from the Quercy fissure fills of France represents a second, longer-legged species known thus far only from the tibiotarsus and tarsometatarsus [10].

Frogmouths have long been classified within the traditional order “Caprimulgiformes” comprising the extant Podargidae, Steatornithidae (oilbird), Caprimulgidae (nightjars and nighthawks), and Nyctibiidae (potoos). However, nearly all recent morphological and molecular phylogenic analyses support the nesting of Apodiformes within a clade uniting the traditional constituents of “Caprimulgiformes”, rendering the latter paraphyletic ([11], [12], [13], [14], [15]; though see [16], [17]). Podargidae are well-supported as part of this larger clade, to which the name Strisores has recently been applied [15]. Unfortunately, the relationships of Strisores to other avian subclades remain unclear. While several morphological analyses have recovered Strisores within a “higher land bird” assemblage including Piciformes, Passeriformes and the traditional constituents of the possibly paraphyletic "Coraciiformes" ([16], [17], [18]), recent molecular phylogenies of Aves placed Strisores within a ‘Metaves’ clade distant from the abovementioned “higher land bird” clades [12], [13], [19], [20]. This disconnect between the phylogenetic signal in morphological and molecular data presents a particular challenge when assigning fossil taxa to extant clades.

The precise position of Podargidae within Strisores also remains uncertain, with recent analyses producing conflicting results, often accompanied by weak statistical support. Morphological phylogenetic analyses have placed Podargidae as the sister taxon to the oilbird *Steatornis caripensis* [16], [17] or as the sister-taxon of Cypselomorphae [14], [15]. In molecular phylogenetic analyses, Podargidae has either been found in a basal polytomy within Strisores [12], [21] or as the sister taxon to Caprimulgidae + Aegothelidae + Apodiformes [13].

In this study, we focus on resolving both the placement of Podargidae within Strisores and relationships among the extinct and extant frogmouth species. As some extinct taxa represent stem lineage frogmouths, explicit definitions of clade names are desirable. We recommend phylogenetic definitions for the taxon names Podargiformes [22] and Podargidae [23]. We recommend restricting the name Podargidae to the frogmouth crown clade. Because the interrelationships of the 13 extant species of Podargidae have not yet been considered in a single analysis, and because a basal divergence within the clade between *Batrachostomus* and *Podargus* + *Rigidipenna* received only modest support [7], it would be premature to attempt to designate two species-level specifier taxa to formulate a node-based definition of Podargidae. At present, we apply Podargidae as the name for the clade including the common ancestor of all living species of *Podargus*, *Batrachostomus*, *Rigidipenna* and all of its descendants. We apply Podargiformes, a name already coined by [22], to the frogmouth total group – all taxa more closely related to Podargidae than to any other extant taxon within Strisores (Steatornithidae, Caprimulgidae, Nyctibiidae or Apodiformes). The designation of Podargiformes to include the crown, Podargidae, and its fossil members is congruent with the recent work on the taxonomic framework of other “family” level clades with few extant members but multiple stem fossil representatives, e.g. Coliidae/Coliiformes [24].

### The Green River Formation and Avian Assemblage

The Green River Formation comprises a suite of Paleogene lacustrine deposits spanning Wyoming, Colorado and Utah. Three major lakes, Lake Uinta, Lake Gosiute, and Fossil Lake, occurred within this system, which during Eocene times was surrounded largely by forested paratropical terrestrial environments [25], [26], [27], [28], [29], [30]. Fossil Lake, from which the specimen described in this paper originates, was the smallest of these major lakes. Fossil Lake has nonetheless yielded more avian taxa than all other Green River localities combined [27]. With the exception of one mass mortality assemblage of the anseriform *Presbyornis* [31], these fossils occur exclusively within the Fossil Butte Member (FBM) [27]. FBM avian fossils are essentially contemporaneous, having been deposited within, at maximum, an interval of a few thousand years [29]. The numerical age of the fossils is close to 51.66±0.09 Ma based on ^40^Ar/^39^Ar dates obtained from a K-spar tuff deposited above the fossiliferous horizons of the FBM [32].

Nineteen species of birds have been described from Fossil Lake [2], [3], [33], [34], [35], [36], [37], [38], [39], [40], [41], [42], [43], [44], [45], [46]. Previously reported fossils, however, provide only a partial picture of the total diversity within the Green River Formation. Many more taxa await formal description, as hinted by the figures and reports of highly complete undescribed fossils that often accompany reviews of the Green River Formation assemblage [27], [47]. Intriguingly, FBM avian taxa evaluated phylogenetically to date have overwhelmingly been placed along the stem lineages of extant avian subclades comprising traditional “orders” or “families” [24], [38], [48], [49], [50].

The new specimen was collected at Thompson Ranch (locality H of [29]), the same locality that yielded the holotype [3]. This locality represents FBM deposits from a nearshore (F-2 deposits of [28]) environment located near the northwest boundaries of the paleoshoreline of Fossil Lake. Thompson Ranch has also yielded the holotype specimens of the lithornithid *Pseudocrypturus cercanaxius* [37] and the enigmatic *Foro panarium* [42], numerous specimens of the stem roller *Primobucco mcgrewi* [38], [51] and the stem frigatebirds *Limnofregata azygosternon* and *Limnofregata hasegawai* [41], [43], as well as many specimens ranging from single bones to complete skeletons currently under study [45]. Fossils from this locality are typically well-preserved and at least partially articulated.

### Institutional Abbreviations

AMNH, American Museum of Natural History, New York, USA; FMNH, Field Museum of Natural History, Chicago, Illinois, USA; NCSM, North Carolina Museum of Natural Sciences, Raleigh, North Carolina, USA; SMF, Forschungsinstitut Senckenberg, Frankfurt, Germany; SMNK, Staatliches Museum für Naturkunde Karlsruhe, Germany; USNM, National Museum of Natural History, Smithsonian Institution, Washington, D.C. USA.

## Results

### Systematic Paleontology

Strisores Baird, 1858 [52]

Podargiformes Mathews, 1918 [22]

*Fluvioviridavis platyrhamphus* Mayr and Daniels, 2001 [3]

#### Assignment to Fluvioviridavis platyrhamphus

**Figure 2:**
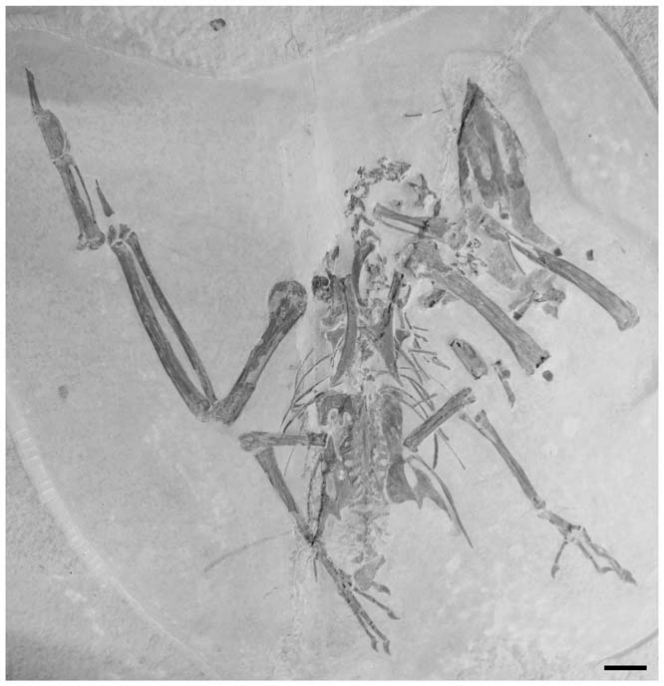
The main slab of the holotype specimen of *Fluvioviridavis platyrhamphus* (SMNK.PAL.2368a) from the Eocene Green River Formation. Scale  = 1 cm.

**Figure 3:**
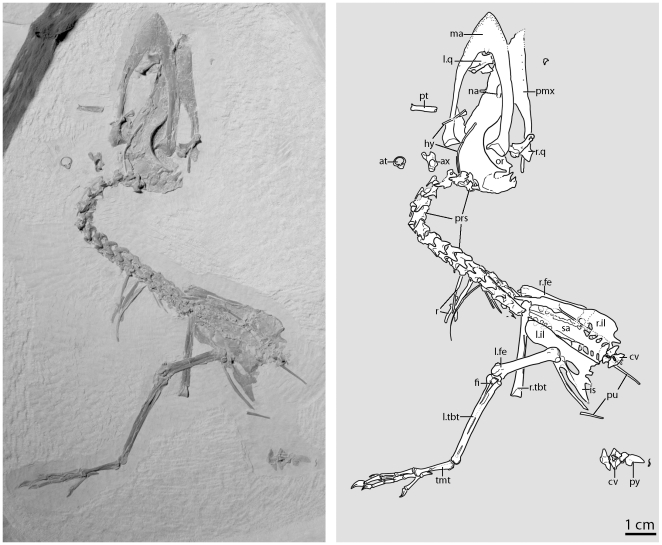
The main slab of the second specimen of *Fluvioviridavis platyrhamphus* (FMNH PA 607) from the Eocene Green River Formation. **Abbreviations:** at, atlas; ax, axis; cv, caudal vertebra; fe, femur; fi, fibula; hy, hyoid; il, ilium; is, ischium; l., left; ma, mandible; na, naris; or, orbit; pmx, premaxilla; prs, presacral vertebrae; pt, pterygoid; pu, pubis; py, pygostyle; q, quadrate; r., right; r, rib; sa, sacrum; tbt, tibiotarsus; tmt, tarsometatarsus.

The partial skeletons SMNK.PAL.2368a+b (Fig. 2) and FMNH PA 607 (Fig. 3) preserve many elements in common and are identical for all comparable morphological character states with the possible exception of a more elongated fibula in FMNH PA 607 (see description). Of the characters listed in the original species diagnosis by Mayr and Daniels [3], both new specimens exhibit an enlarged head with a wide, dorsoventrally flattened beak, and a strongly abbreviated tarsometatarsus. Additional diagnostic characters of the pectoral girdle and forelimb were listed by Mayr and Daniels [3], but unfortunately those portions of the skeleton are not preserved in FMNH PA 607. Other morphological characters held in common are highlighted in the description below.

FMNH PA 607 is approximately 85% the size of SMNK.PAL.2368a+b. The ratios among and between the skull and long limb elements are nearly identical. For example, the upper beak is about the same length as the tibiotarsus in both specimens. Slight differences in ratios are within the range of error when measuring crushed specimens; however, the tarsometatarsus is slightly smaller than expected (75% not 85%) relative to the other measurements comparing the two specimens. The pes of FMNH PA 607 has similar portions to that of SMNK.PAL.2368a+b. Significant size dimorphism occurs between males and females in *Batrachostomus*, but no reports of size dimorphism exist for *Podargus* (Holyoak 1999).

Here we consider both SMNK.PAL.2368a+b and FMNH PA 607 to represent the same species. In addition to the morphological character combinations in common detailed above and in the description, both SMNK.PAL.2368a+b and FMNH PA 607 are from the same locality (Thompson Ranch, locality H). All of the primary fossiliferous horizons of the Fossil Butte Member are hypothesized to sample an interval restricted to a few thousand years or less [29]. The only prospective basis for naming a new taxon would be slight differences in overall size and the relative length of the tarsometatarsus. Naming a new taxon based on these criteria we believe would be premature given currently poor understanding of size variation, dimorphism, and morphological variation in *Fluvioviridavis* and even extant Podargidae. However, each specimen of *Fluvioviridavis* is scored separately in the phylogenetic analysis (see below) to allow for this possibility. The amended diagnosis presented below is based on both specimens.

#### Amended diagnosis

*Fluvioviridavis platyrhamphus* differs from all other Aves by the following combination of character states: dorsolaterally oriented naris that is ventrally bordered by a thin sheet of bone (character 2∶1); large skull with dorsoventrally compressed beak (character 3∶1); fully ossified palate with a ventrally directed choana framed by ventrally projecting lamina (character 15∶1); mandible straight in lateral view (character 33∶1) and concave lingually and convex ventrally (character 35∶1); small posteriorly directed projection at the posterior edge of the mandibular symphysis*; sternum with two pairs of shallow notches; short humerus with enlarged proximal portion; manual digits 1 and 2 with small claws; and, an extremely abbreviate tarsometatarsus shorter than half the length of the carpometacarpus (character 85∶1). Asterisk denotes autapomorphy as optimized in the analysis.

*Fluvioviridavis platyrhamphus* can further be differentiated from proposed closely related taxa. It is differentiated from *Prefica nivea* by the presence of a wider mandibular symphysis and a more anteroposteriorly elongated sternum. A more pointed beak that is not strongly decurved and a smaller ring of sclerotic plates differentiate *Fluvioviridavis platyrhamphus* from *Masillapodargus longipes*. *Fluvioviridavis platyrhamphus* differs from all extant species of Podargidae by the absence of a distinct nasofrontal hinge (character 7∶0), presence of a pointed beak tip, and the absence of lacrimal "horns" projecting posteriorly at front of orbit (character 9∶0). *Fluvioviridavis platyrhamphus* differs from *Quercypodargus olsoni* in having a much more abbreviate tarsometatarsus (character 85∶1) and from *Eurofluvioviridavis robustipes* in having a fully ossified palate and a midline plantar crest on the tarsometatarsus.

#### Measurements

Skull, maximum length  = 50.7; Pelvis, length from preacetabular ilium to spina dorsolateralis ilii  = 28.5; Pelvis, length from preacetabular ilium to end of pubis  = 38.2; Femur  = 21.1; Tibiotarsus  = 28.8; Tarsometatarsus  = 11.9; Phalanx I-1  = 5.5; Phalanx I-2  = 3; Phalanx III-1  = 5.1; Phalanx III-2  = 4.6; Phalanx III-3  = 4.7; Phalanx III-4  = 3.9; Phalanx IV-1  = 4; Phalanx IV-2  = 2.9; Phalanx IV-3  = 3.1; Phalanx IV-4  = 3.7; PhalanxIV-5  = 2.7.

### Description

#### Skull

**Figure 4:**
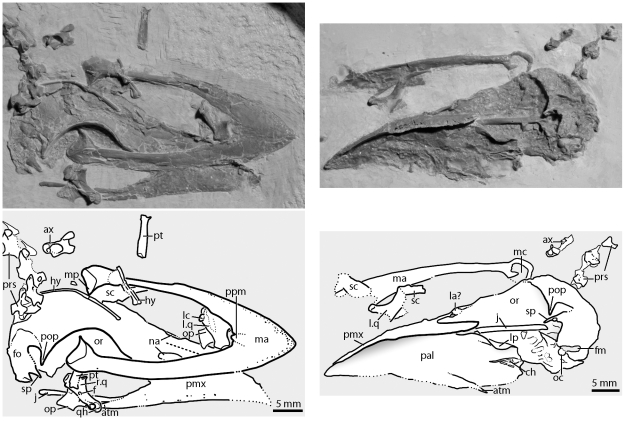
The skull of *Fluvioviridavis platyrhamphus* (FMNH PA 607) in the main slab (left) and the counter slab (right). **Abbreviations:** atm, angulus tomialis maxillaris; ax, axis; ch, choana; f, foramen; fm, foramen magnum; fo, fossa; hy, hyoid; il, ilium; is, ischium; j, jugal bar; l., left; la, lacrimal; lc, lateral condyle of quadrate; lp, lateral process; ma, mandible; mc, medial cotyle; mp, medial process; na, naris; oc, occipital condyle; op, orbital process; or, orbit; pal, palate; pmx, premaxilla; pop, postorbital process; ppm, posterior process of the mandible; prs, presacral vertebrae; pt, pterygoid; q, quadrate; qh, quadrate head; r., right; sc, sclerotic ossicle; sp, squamosal process.

The skull of FMNH PA 607 is exposed in both dorsal and ventral view, but strongly compressed dorsolaterally (Figs. 3, 4). The cranium is thus exposed in right dorsolateral view with the rest of the preserved skeleton in the main slab. The reverse side of the skull was prepared separately exposing details of much of the palate, orbital region, and braincase. The unusually large skull is equivalent to ¾ the length of the presacral vertebral column. The size of the head relative to the body in both FMNH PA 607 and the holotype is proportionally similar to that of extant *Podargus* and Coraciidae (rollers).

The skull is well ossified and few sutures can be discerned among the anterior skull elements. The lateral sides of the beak of FMNH PA 607 converge anteriorly to form a narrow slightly decurved tip, as in Podargidae. The lateral edge of the beak curls ventrally to form a thin cutting surface. Abundant small foramina and slightly pronounced ridges ornament the outer surface of the maxilla as in Podargidae and many other birds with large beaks. The rostrum accounts for approximately half the total length of the skull and the anteroposteriorly elongated and narrow external naris is located well posterior of the tip of the beak. The nares are separated by a wide internarial bar as in SMNK.PAL.2368a+b. A clear midline suture between the frontal processes of the premaxillae is present in both specimens of *Fluvioviridavis* (Fig. 4; Figure 1 of [4]). The orientation of the long axis of the external naris cannot be precisely determined from FMNH PA 607 because of the crushing of the specimen, but appears to be dorsolateral in SMNK.PAL.2368a+b. The posterior margin of the narial opening is located close to the anterior margin of the orbit. The a sharp anterolateral ( = anterior in other Aves) margin of the external naris extends anterolaterally onto the dorsal portion of the beak in SMNK.PAL.2368a+b as in *Podargus* (Figure 1 of [4]). In *Steatornis* and Coraciidae, this transition from the anterior margin of the external naris is rounded and is not overhanging the anterior end of the external naris. This lip of bone overhangs the ventral portion of the external naris, and here the bone is thin and has a “honey-comb” pattern. This feature is only present in the holotype of *Fluvioviridavis,* Podargidae, and some rollers [53] among surveyed birds. The posterior portion of the maxilla tapers posteriorly and terminates in a distinct extension posterolateral to the contact with the jugal bar ( = angulus tomialis maxillaris of [16]). This character state is only present in *Podargus* and *Steatornis* within Strisores, though it is also see within some other clades (e.g., Anseriformes, Galliformes) ([16]: Character 408). A conspicuous, lineate nasofrontal hinge seems to be absent. The jugal bar is straight in lateral view and is rounded in cross-section.

There is no division between the antorbital fenestra and the orbit. A well-developed lacrimal is lacking in both FMNH PA 607 and SMNK.PAL.2368a+b and it is possible this element was absent, although a small ossification on the dorsolateral margin of the anterior portion of the orbit may represent a highly reduced lacrimal in FMNH PA 607 (Fig. 4). Alternatively, this small projection may represent a precursor to the lacrimal “horn” at the anterodorsal margin of the orbit as in *Podargus* ( = processus dorsolateralis nasalis of [16]). The dorsolateral margin of the orbit is raised relative to the midline and the interorbital distance is wide. The frontal and parietal sutures appear to be closed as in most Neognathae. The interorbital septum appears to be imperforate. A small optic nerve foramen is visible, and the small cone-like protrusion bounding the foramen found in Caprimulgidae and Nyctibiidae [14], [15] is absent. The postorbital process and the zygomatic process do not touch and both taper ventrally to terminate in a point (Fig. 4). The temporal fossa is distinctly rimmed and approaches the midline as in *Batrachostomus* and *Podargus.* The depth of the fossa in *Fluvioviridavis* is more similar to that of *Batrachostomus*, whereas it is shallower in *Podargus*. Details of the tympanic region are difficult to discern. The lateral projection of the paroccipital process forms a broad posterior wall to the tympanic region. The occipital condyle is located on the posteroventral portion of the skull. Crushing and displacement in the back of the skull precludes easy identification of foramina in the basioccipital region. The parasphenoid rostrum extends dorsal to the palate but the anterior portion is covered by other elements (Fig. 4). Basipterygoid processes are not present.

#### Quadrate

Both quadrates are preserved but rotated out of life position. The left quadrate is exposed in anteromedial view, whereas the right quadrate is exposed in anterolateral view (Fig. 4). The main body of the quadrate is relatively straight with little bowing of the posterior edge. Distinct vertical ridges on both the lateral and medial sides of the main body separate it from the orbital process. The orbital process of the quadrate is squared off at its anterior margin and reduced like that of Caprimulgidae, Nyctibiidae, and Aegothelidae [14], [15]. This is identical in SMNK.PAL.2368a+b [3]. A pneumatic foramen perforates the middle of the main body in anteromedial view. The squamosal capitulum is much larger and more dorsally expanded than the otic capitulum and the two heads are separated by a weakly developed intercapitular groove. The left quadrate preserves a large tuberculum subcapitulare (sensu [54]).

The mandibular process of the quadrate expands anterolaterally and medially. The medial side bears a well-projected pterygoid condyle. The morphology of the pterygoid and of its articulation surface on the quadrate indicate that these had limited contact given the small area of the articular surface of both elements. This conformation is more similar to that of rollers (e.g., *Brachypteracias leptosomus*, FMNH 384731) than that of nightjars (*Caprimulgus carolinensis*, NCSM 18510). The poorly developed and indistinct articular surface for the quadratojugal is located just dorsal to the lateral mandibular condyle (Fig. 4). The lateral mandibular condyle is more dorsally oriented than that of the medial mandibular condyle and is distinctly mediolaterally compressed. The distal surface of the lateral mandibular condyle is slightly concave anteroposteriorly whereas the distal surface of the medial mandibular condyle is distinctly rounded (Fig. 4). A similar morphology of the distal end of the quadrate is uniquely present in frogmouths among living birds and was listed as an autapomorphy of Podargidae by Livezey and Zusi ([16]).

#### Palate

The palate of FMNH PA 607 is well exposed in ventral view (Fig. 4). Fused premaxillae, maxillae, and palatines form an extensive secondary palate as in Podargidae and *Balaeniceps rex* (the Shoebill). It is not clear if the vomers are also incorporated into the palate, located dorsally, or are entirely absent. In frogmouths, small grooves and crenulations decorate the surface of the secondary palate. The palatines are fused both posteriorly and anteriorly to the choana. Fusion of the palatines anterior and posterior to the choana is extremely rare in Aves but is also found in *Steatornis* and *Balaeniceps* ([16]: Character 442). The absolute size of the choana in the fossil is small, but the exact proportions cannot be obtained because of the distortion. Thin lamella lateral to the choana outline the opening and are more prominent posteriorly than anteriorly. It is not clear if the choana is divided by a lamina on the midline as in Steatornithidae. The palatines are not expanded laterally, in contrast to the condition in Podargidae, Caprimulgidae, Nyctibiidae, Aegothelidae, and Apodiformes. The lateral margins of the palatine are folded ventrally as a result of crushing of the specimen. A fossa lies between the lateral side of the palatine and the lamina ventral to the choana.

The left pterygoid is disarticulated (Fig. 4) and is exposed in ventral view. This straight element is slightly anteromedially expanded. The posterior end has a concave facet for articulation with the pterygoid condyle of the quadrate. The anterior end simply contacts the palatine.

#### Mandible

The entire ventral surface of the mandible is exposed on the main slab whereas the right ramus is preserved in dorsal view on the reverse-prepared counter-slab (Fig. 4). The dorsolateral edge of the anterior half of the mandible is covered in abundant tiny foramina. The mandibles meet at an anteroposteriorly expanded symphysis that comprises slightly less than 1/3^rd^ the total mandibular length as in *Podargus*. As with the dorsal portion of the skull, the anterior end of the mandible terminates in a distinct point, whereas in Podargidae it ends in a rounded edge. A distinct posteriorly projecting process located on the posterior edge of the symphysis and framed by shallow grooves is an autapomorphy of *Fluvioviridavi*s (Fig. 4: ppm). The anterior portions of the rami are mediolaterally expanded as in *Podargus*. In dorsal view, the right ramus has a pronounced, monotonic curvature “producing continuous lateral concavity” ([16]: Character 658). This character state is present in *Podargus* as well as several distantly related broad beaked taxa such as *Balaeniceps* and *Cochlearius* [16]. The rami appear straight from their entire length in lateral view. However, given that the specimen is preserved in dorsal and ventral views, slight curvature or decurvature cannot be ruled out. Straight rami are rare among Aves but do occur in extant Podargidae.

The articular region of the mandible is well preserved and exposed in dorsal and ventral views. In ventral view, the articular area expands medially to form a distinct medial process that appears not extend dorsal to the rest of the rami. The medial process tapers medially and terminates in a small tuber that is slightly angled posteriorly. A weakly rimmed fossa is located on the posterior margin of the articular area between the termination of the medial process and the lateral side of the ramus. There is no retroarticular process; the mandible terminates just posterior to the articular facets with the quadrate. The lateral edge is rounded and continuous with the posterior border of the mandible. This rim circumscribes a deep fossa that articulated with quadrate. The exact depth of the medial cotyle of the mandible cannot be ascertained. Therefore, it is unclear whether FMNH PA 607 shared the jaw locking mechanism formed by a deep medially opening fossa fitting into an enlarged medial condyle, which is unique to Podargidae among extant birds [16]. The anterior part of the articular area buttresses the lateral portion of the medial cotyle.

#### Hyoid

The hyoid is incompletely preserved. The ceratobranchials lie on the dorsal surface of the skull roof. Although the hyoid partially wraps around the skull in some extant birds (e.g., Trochilidae and Picidae), the displacement of the mandible suggests it is not in life position in the fossil.

#### Sclerotic ossicles

Fragments of large sclerotic ossicles are disarticulated from life position and are located near the left side of the mandible. The ossicles are large (2.7 mm X 5 mm) as in other nocturnal ‘Caprimulgiformes,’ but are relatively smaller than those of Podargidae and *Masillapodargus* (SMF ME 3405a).

#### Vertebrae

The vertebral column is preserved, and nearly all the vertebrae are articulated except for the atlas, axis, and part of the caudal series (Fig. 3). There are nineteen presacral vertebrae with no visible gaps with the exception of the disarticulated axis and atlas. Nineteen presacral vertebrae are present in Steatornithidae and *Protocypselomorphus*, whereas Podargidae and other Strisores possess 18 or fewer [55].

The atlas, preserved in anterior view, bears a large condyloid fossa that occupies all of the body of the element (Fig. 3). A thin rim circumscribes the fossa for articulation with the occipital condyle and an open incisure ( = incisura fossae of [56]) receives the odontoid process of the axis. The axis is anteroposteriorly similar in length to the third cervical vertebra. The prezygapophyses of the axis are slightly anteriorly and laterally expanded. The postzygapophyses are club-shaped and separated from the neural spine by a slight groove. The neural spine is also club-shaped.

At least presacral vertebrae 3–5 bear small keel-shaped ventral processes on their midlines; the ventral surface is not exposed in the other presacral vertebrae. Only the third presacral vertebrae possesses an osseous bridge from the transverse process to the postzygapophyses ([18]: character 52). A deep midline fossa between the postzygapophyses is present in most presacral vertebrae as observed in SMNK.PAL.2368a+b [3]. The prezygapophyses of the cervical vertebrae are laterally expanded as robust and rounded structures as in *Caprimulgus carolinensis* (NCSM 18510). Small posterodorsally oriented epipophyses lie on the dorsal surface of postzygapophyses. The neural spines of the presacral vertebrae are low anteriorly and expand dorsally in the more posterior presacral vertebrae. The last four presacral vertebrae have ossified aponeuroses on the neural arches and on the lateral portions of the transverse processes (Fig. 3). A notarium is absent.

The sacral vertebrae are exposed in dorsal view. At minimum twelve vertebrae (four vertebrae with transverse processes anterior to the mid-sacral series, four vertebrae in the mid-sacral series with inconspicuous diapophyses, and four vertebrae with transverse processes posterior to the mid-sacral series) are incorporated into the synsacrum. The synsacral spine is high anteriorly and shortens posteriorly reaching the same level as the sacral ribs halfway along the sacrum. The more anterior sacral ribs are laterally directed whereas the more posterior ribs slant posterolaterally. The widest sacral vertebrae are located at the midpoint of the sacrum just medial to the level of the acetabula. Small gaps between the ilium and the synsacrum ( = foramina intertransversia) are present for much of the length of the ilia. The disarticulation of the sacrum from the ilium appears to be not purely a consequence of taphonomy, indicating that the elements were not fully co-ossified. This character state is also present in Podargidae and *Steatornis*.

Seven vertebrae compose the caudal series (Fig. 3). The anterior three are articulated with the sacrum and exposed in dorsal view. The posterior four caudal vertebrae are articulated with the pygostyle and are exposed in ventral view. The transverse processes of the first four caudal vertebrae project laterally, whereas the transverse processes of caudal five project posterolaterally. The transverse processes of the sixth caudal vertebra project anterolaterally. The last sacral vertebra has the longer transverse processes than any part of the caudal series. The last free caudal is reduced and partially co-ossified to the pygostyle (as in *Caprimulgus*). The fifth caudal vertebra preserves a well-developed haemal process whereas the fourth caudal vertebra clearly does not preserve one. The mediolaterally compressed pygostyle (Fig. 3) appears to be incompletely preserved but is overall similar in morphology and size to that of SMNK.PAL.2368a+b.

#### Pelvis

**Figure 5:**
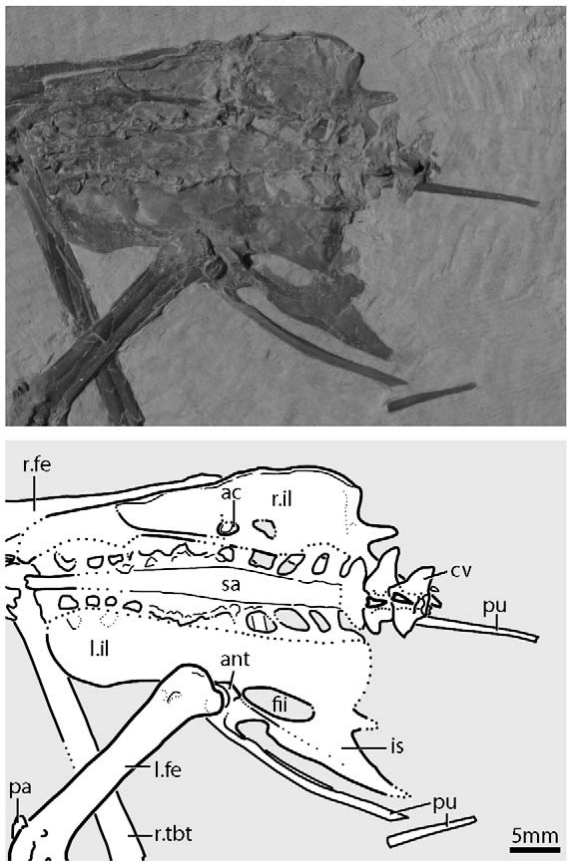
The pelvis of *Fluvioviridavis platyrhamphus* (FMNH PA 607) on the main slab in dorsolateral view. Abbreviations: ac, acetabulum; ant, antitrochanter; cv, caudal vertebra; fe, femur; fi, fibula; fii, foramen ilioischiadicum; il, ilium; is, ischium; l., left; pa, patella; pu, pubis; py, pygostyle; r., right; sa, sacrum; tbt, tibiotarsus; tmt, tarsometatarsus.

The well-preserved pelvis lies on its right side such that the left side is in dorsolateral view (Figs. 3, 5). The pelvis appears identical to the pelvis of SMNK.PAL.2368a+b in its substantial width and in all preserved morphologies.

The left ilium is preserved in lateral view whereas the right ilium is preserved in medial view (Fig. 5). The pre- and postacetabular portions of the ilia are similar in length measuring from the middle of the acetabulum. The preacetabular portion is concave laterally and rounded anteriorly in lateral view. Its dorsal margin appears to serve as the attachment site for the sacral ribs. The preacetabular portions of the ilia do not meet on the midline and are well separated. A dorsal ilia crest is not developed. In medial view, there is a distinct ridge originating in the middle of the preacetabular portion of the ilium that stretches posteriorly to form the ventral margin. The body of the antitrochanter projects posterodorsally beyond the dorsal rim of the acetabulum.

The postacetabular process of the ilium fuses to the ischium posteriorly to enclose an oval ilioischiadic foramen. A slight longitudinally oriented ridge marks the contact between the ischium and the ilium. A distinct posteriorly directed process is also located on the posterior contact between the ischium and the ilium. A similar process is found in a variety of taxa including “Caprimulgiformes” and Coraciidae. Distinct dorsolateral iliac spines are present on the postacetabular portion of the ilium; a state also present in SMNK.PAL.2368a+b and *Batrachostomus* [3].

The ischium posteriorly terminates in an acute point (Fig. 5). A small obturator process is present but does not appear to contact the pubis. The ventral portion of the ischium and pubis lie parallel in both FMNH PA 607 and SMNK.PAL.2368a+b. The pubis appears to lack a preacetabular process. The shaft of the pubis is straight, bears a low elongated ridge parallel to its ventral border, and extends far past the posterior end of the ischium. The posterior portion of the pubis tapers slightly and has a blunt distal end.

#### Hind limb

The left hind limb is complete whereas the right includes the femur and tibia (Fig. 3). The left femur is exposed in posterior view and the most of the right is obscured by the right ilium. The posterolateral side of the femur is marked by small foramina and grooves. The trochanteric crest is weakly projected and does not extend much beyond the dorsal margin of the femoral head. The femoral shaft is straight. The intercondylar sulcus is poorly developed. The tibiofibularis crest is robust, and the adjacent lateral condyle is thin and pointed posteriorly. A large tubercle for the m. gastrocnemius attachment lies just dorsal to the lateral condyle. A small, rounded patellar ossification is preserved on the anterior side of the femur (Fig. 5).

The well-preserved left tibia is exposed in medial view, whereas the right is preserved in anterior view. The proximal surface is broadly convex. The lateral cnemial crest terminates in a blunt end and does not extend proximal to the condylar articular surfaces. The anterior cnemial crest also has a limited distal extent. It has a rounded profile in lateral view, and is weakly anteriorly projected as in *Steatornis,* Podargidae and Coraciidae. The robust fibular crest is asymmetrical in anterior view; the distal portion is more laterally expanded than the proximal portion. The lateral distal condyle is weakly expanded anteriorly relative to the shaft. The lateral surface of this condyle is slightly concave. In posterior view, the trochlear surface is shallow. The posterodistal ends of the trochlear crests are rounded and converge proximally in posterior view.

In lateral view, the head of the fibula is asymmetrical; the posterior portion is more expanded than the anterior portion. A deep pocket is located on the lateral side of the proximal portion. Parts of the fibular shaft can be traced for the length of the tibia in FMNH PA 607, but the fibula appears to be much shorter in SMNK.PAL.2368a+b. A rounded ridge on the lateral side of the distal end of the tibia marks the termination of the fibula in FMNH PA 607, and this feature does not seem to be visible in SMNK.PAL.2368a+b.

**Figure 6:**
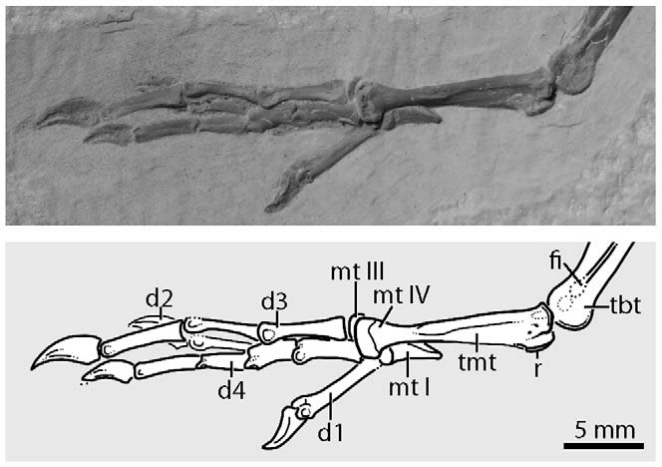
The left pes of *Fluvioviridavis platyrhamphus* (FMNH PA 607) on the main slab in lateral view. **Abbreviations:** d#, digit number; fi, fibula; mt#, metatarsal #; r, ridge; tbr, tibiotarsus; tmt, tarsometatarsus.

The tarsometatarsus is preserved in lateral view (Fig. 6). The element is very abbreviated (20% the length of the femur + tibiotarsus + tarsometatarsus) as in *Prefica nivea*, *Steatornis*, and *Eurofluvioviridavis*. There are at least two short hypotarsal crests, of which the medial crest is more projected and dorsally elongate. It is not clear if hypotarsal canals are present. There is a distinct groove on the lateral side of the tarsometatarsus located 1/3 the length of the element from the distal end. The plantar surface of the tarsometatarsus bears a midline plantar crest. Trochlea IV is exposed on the surface and appears to have at least a small wing-like flange, but there is no evidence for facultative zygodactyly. Trochlea III is barely exposed behind trochlea IV, and extends further distally. The attachment site for metatarsal I is obscured. Metatarsal I itself is about one-third the length of the tarsometatarsus and does not show significant distal expansion.

The well-preserved left pes is clearly anisodactyl (Fig. 6). As in the holotype (SMNK.PAL.2368a+b), the third digit is the longest, and the second digit is shortest. The first phalanx of digit one is the longest of all the phalanges as in most birds with an unreduced hallux. The unguals are relatively short, similar in overall length, weakly recurved, bear lateral sulci, and have small flexor tubercles.

### Phylogenetic analysis

#### Taxon Sampling

In order to reassess the phylogenetic position of *Fluvioviridavis* taking into account the data from the new specimen, we included this taxon in a data matrix including fossil and extant taxa of Strisores (Apodiformes + ‘Caprimulgiformes’). The only previous analysis of the relationships of that taxon, based on a composite supraspecific terminal, recovered very weak support for the Fluvioviridavidae as the sister taxon to a heterogeneous clade of “higher land birds” including Psittaciformes, Piciformes, Passeriformes, Cuculiformes, Leptosomidae, Podargidae and the traditional contents of "Coraciiformes" [4]. Later affinities to Strisores were suggested [5]. Although few character states support unambiguous placement of *Fluvioviridavis* within a specific avian clade, a suite of character states in *Fluvioviridavis* and in combination with current knowledge of avian character state distribution, indicated that the taxon fell near or within Strisores. For example, *Fluvioviridavis* processes an abbreviated orbital process of the quadrate as in Strisores. Other shared character states between *Fluvioviridavis* and Strisores are described below. Assessment of the character data in the fossil was determined to support Strisores affinities and thus this dataset was developed.

Supergeneric terminal taxa of Mayr [15], [55] were replaced by nine species-level exemplar taxa for Steatornithidae, Caprimulgidae, Nyctibiidae, Aegothelidae, Apodidae, Hemiprocnidae, and Trochilidae in order to avoid problems associated with composite higher-level taxa and facilitate inclusion of molecular sequence data. Species-level exemplar taxa were also used for all other clades included in the analysis. Proposed extinct representatives of Strisores including *Masillapodargus longipes*, *Prefica nivea*, and *Paraprefica kelleri* were included to sample morphology from potential stem group members of major subclades within Strisores (i.e., Podargidae, Steatornithidae, Nyctibiidae). Both specimens of *Fluvioviridavis* were scored independently in the phylogenetic matrix and an additional terminal including the combined scorings was created, so that analyses could be run both treating the specimens separately and as representatives of a single species. Overlapping scorings were identical for the two specimens.

Given the uncertainty surrounding the closest extant relatives of Strisores, we included multiple outgroup taxa representing nearly all proposed sister taxa for Strisores or subclades within the group [13], [15], [18], [55], [57] as well as Tinamiformes. Four distinct outgroup taxa were scored from exemplar species of Tinamiformes (*Crypturellus undulatus*), Trogonidae (*Trogon massena*), Eurypygidae (*Eurypyga helias*), and Leptosomidae (*Leptosomus discolor*) for reasons explained below. We follow Mayr [14], [15], [55] in selecting a tinamou as a distal outgroup comparison for Strisores. This choice is the most conservative because there is overwhelming molecular (e.g., [13]) and morphological evidence [16], [17], [18] that tinamous lie outside Neognathae and because there is little consensus concerning basal neoavian relationships. Neognath taxa were selected based on previous hypotheses of the relationships of Podargidae and/or Strisores. Trogons were hypothesized to be closely related to Steatornithidae by Mayr [57] although this relationship has not been found in any large molecular analyses. We chose the sunbittern *Eurypyga* as an exemplar of ‘Metaves’ as Strisores was recovered as part of this clade by Fain and Houde [19] and Hackett et al. [13]. We also selected the cuckoo-roller *Leptosomus* for outgroup comparison given that a close relationship between this taxon and Strisores was recovered by Mayr [58] and Mayr et al. [59], though we note that the cuckoo-roller is distantly related to Strisores in the results of recent molecular analyses [12], [13]. Furthermore, the four chosen outgroups have a different suites of presumably plesiomorphic character states. For example, *Eurypyga* and *Leptosomus* have a cup-like scapular articulation of the coracoid and a supracoracoid nerve foramen whereas both of these features are absent in *Crypturellus undulatus* and *Trogon massena*. The results of all four analyses were then compared.

#### Morphological Data

The assembled matrix combines characters from morphological datasets by Mayr and Clarke [18], and Mayr [15], characters proposed as apomorphies of Podargidae by Livezey and Zusi [16], [17], and newly described characters. A total of 99 morphological characters were included, of which two (characters 27 and 64) were considered ordered as proposed by Mayr [55]. Specimens examined, character descriptions with citations, and scorings are included in the Information S1. All extant taxa and the fossil taxa *Fluvioviridavis* and *Prefica* were scored by directed observation. For *Masillapodargus* and *Paraprefica* we relied on the images and descriptions of Mayr [8], [55], [57].

A set of soft-tissue characters (characters 100-108) were scored following Mayr [15] in order to include all available character sets. Family level scorings were provisionally accepted for the species-level taxa used in this paper. However, because the scorings were not based on the species-level, the results were excluded from the primary combined analysis. We included these scorings in an additional analysis to test their potential effects. The complete morphological dataset (osteological and soft-tissue) is available at Morphobank (morphobank.org: Project 332).

#### Molecular Data

Nucleotide sequence data for one mitochondrial (*cytochrome b*) and two nuclear (*c-myc* exon 3 and *RAG-1*) gene regions were obtained from GenBank. Accession numbers and original citations are in the Information S1. Sequences were aligned in ClustalX 1.83 and the preferred alignment was visually inspected and adjusted manually. All sequences were then concatenated and appended to the morphological data matrix for combined analysis.

#### Analysis

The combined dataset was subjected to parsimony analysis in PAUP*4.0b10 [60] using the Branch and Bound algorithm. Alternate analyses with all characters unordered were also conducted. Branches with a minimum length of 0 were collapsed (i.e., rule 1 of [61]). Bootstrap support was calculated from 1,000 replicates using a heuristic search strategy with random taxon addition sequence and TBR branch swapping. Trees were rooted separately with each of the four suggested outgroups (see above) to explore the effects on character optimization of different proposed positions for Strisores within Aves.

## Results

**Figure 7:**
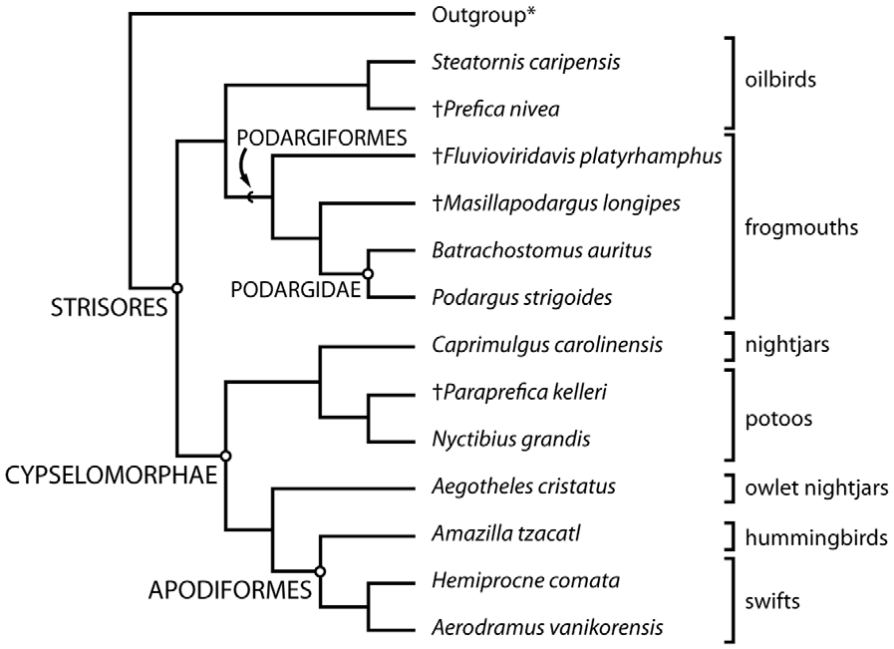
The relationship of *Fluvioviridavis platyrhamphus* to that of other Strisores using a combined dataset (TL = 2119, CI = 0.713, RI = 0.439) and a morphology-only dataset (TL = 175, CI = 0.600, RI = 0.700). See text for outgroup selection(s). Circles  =  nodes, chevrons  =  stem-groups.

Analyses of the combined dataset and the morphology-only dataset yielded trees with the same topology (Fig. 7). The combined dataset produced a unique tree (TL = 2119, CI = 0.713, RI = 0.439) when *Crypturellus undulatus* was used as the sole outgroup. Additional analyses utilizing the three alternative outgroups each resulted in a single tree with an identical topology to that recovered in the initial analysis, though length and some indices varied as follows: *Trogon massena* (TL = 2195, CI = 0.721, RI = 0.446), *Leptosomus discolor* (TL = 2133, CI = 0.715, RI = 0.444), and *Eurypyga helias* (TL = 2006, CI = 0.741, RI = 0.471). An analysis including only morphological characters also resulted in a unique tree (TL = 177, CI = 0.599, RI = 0.699) using *Crypturellus undulatus* as an outgroup. Finally, the inclusion of muscle and soft-tissue characters scored by the “family” level by Mayr [15] to the morphology-only analysis increased the tree length but did not affect topology (TL = 188 CI = 0.606 RI = 0.700). Treating the two ordered characters as unordered also did not affect topology, though tree length decreased by four steps when compared to the combined dataset. Given the congruence of the trees from analyses using all datasets, outgroups and ordering strategies, we treat all results together below.

Strisores was found to be monophyletic, which is congruent with the morphological analyses of Mayr [14], [15] and Livezey and Zusi [16], [17] and molecular-sequence based analyses of Ericson et al. [12] and Hackett et al. [13]. Cypselomorphae are also found to be monophyletic in congruence with Mayr [14], [15] and Hackett et al. [13]. Outgroup choice did not affect the relationships of Strisores in both the morphological-only and combined analyses, although support varied near the base of Cypselomorphae and Steatornithiformes + Podargiformes.

*Fluvioviridavis* was recovered within Strisores at the base of a clade containing Podargidae (*Podargus* + *Batrachostomus*) and *Masillapodargus* (Fig. 7). Our results support placement of *Fluvioviridavis* and *Masillapodargus* outside Podargidae as stem lineage members of Podargiformes. Monophyly of Podargiformes is supported by multiple cranial synapomorphies including: a rim surrounding the external naris that extends anterolaterally onto the dorsal surface of the beak (character 2∶1), the absence of pterygoid-basipterygoid contact (character 22∶2), a well rounded posterior portion of the articular portion of the mandible (character 32∶1), and virtually straight ventral margin of the mandibular rami (character 33∶1), and monotonic curvature of the mandibular rami producing continuous lateral concavity (character 35∶1). No postcranial characters unambiguously support the placement of *Fluvioviridavis* within Podargiformes. *Masillapodargus* is supported as crownward of *Fluvioviridavis* based on greatly enlarged sclerotic ossicles (character 10∶1), a rounded anterior portion of the mandible symphysis (character 34∶1), and a straight scapular blade with a ventrally directed tip (character 51∶1).

Character data supporting placement of *Fluvioviridavis* within Podargiformes are concentrated in the skull. Recognizing potential postcranial synapomorphies for Podargiformes and/or Strisores is difficult given the current lack of consensus regarding the extant sister taxon of Strisores and complex homoplastic character distributions in landbird clades. Several of the derived cranial characters shared by *Fluvioviridavis* and Podargidae are also seen in the much larger *Balaeniceps* (Shoebill) and *Cochlearius* (Boat-billed Heron). These taxa also share a greatly enlarged bill, suggesting correlation between expansion of the beak and some palatal characters. However, the postcranial skeletons of these aquatic-feeding birds are extremely dissimilar to that of *Fluvioviridavis*. At present no convincing evidence exists for close relationship between *Fluvioviridavis* and any clade outside Strisores.

Character states recovered as unambiguously optimized synapomorphies of the crown clade Podargidae include: lacrimal "horns" projecting posteriorly at front of orbit (character 9∶1); articular facet for the scapula of the coracoid flat (character 47∶1); and very long lateral trabeculae and short intermediate trabeculae of the sternum (character 57∶1). The first and third character states likely represent unique autapomorphies of Podargidae whereas a flat articular facet for the scapula of the coracoid has evolved independently in many disparate avian clades. Additional character states optimized as synapomorphies of Podargidae under DELTRAN include: a distinct nasofrontal hinge (character 7∶1), strongly protruding posterolaterally directed processes of the palatine (character 14∶1), a posteriorly opening internal choana (character 16∶1), a fossa on the ventral surface of the palatine that is anterior to the internal choana and is separated from its counterpart by a midline lamina (character 21∶1), and interlocking mechanism between the quadrate and articular portion of the mandible (character 31∶1). Because of missing data, it is currently uncertain whether these character states are synapomorphies of Podargidae or the clade *Masillapodargus* + Podargidae. Characters states 16∶1 and 21∶1 appear to be unique to Podargidae within Aves. The enlarged posterolateral processes of the palatine (character 16∶1) appear to be acquired independently in Podargidae and Cypselomorphae given the absence in *Fluvioviridavis* and *Steatornis*.

Here we recover a relationship between Podargiformes and Steatornithiformes exclusive of all other Strisores. This relationship is supported by a number of character states but given that these clades are basal within Strisores and some relevant characters are homoplastic within Aves, the optimization of character states are likely to change with outgroup choice. However, multiple character states were consistently optimized as synapomorphies of the clade Podargiformes + Steatornithiformes regardless of which of the four alternate outgroups was used in our analyses. These character states include: dense neurovascular pitting on the rostrum (character 5∶1), posterior termination of the maxilla extending laterally and posteriorly of the contact with the jugal bar ( = angulus tomialis) (character 6∶1), ventrally projecting lamina framing a closed choana (character 15∶1) (absent in Podargidae but present in both *Fluvioviridavis* and *Masillapodargus*), palatine fused anterior to the internal choana (character 17∶1), temporal fossae meeting or almost meeting at midline of skull (character 26∶1), and anterior face of manual phalanx II-1 dorsoventrally widened, giving phalanx a T-shaped cross section (character 71∶1). A cup-like scapular articulation of the coracoid is present in both stem members of Podargiformes (*Fluvioviridavis*) and Steatornithiformes (*Steatornis*) and is likely plesiomorphic for the group as it is for Aves (see above). A sister group relationship between Podargidae and Steatornithidae was also recovered in the morphological analysis of Livezey and Zusi [16], [17] and the Bayesian analysis of the β-fibrinogen intron 7 sequence of Ericson et al. [12] (though note this clade was not supported in analysis of the combined five gene dataset).

The morphological analysis of Mayr [14], [15] found *Steatornis* and Podargidae as successive sister-taxa of Cypselomorphae. A clade uniting Podargidae + Cypselomorphae to the exclusion of *Steatornis* was supported by two unambiguous character states: enlarged posterolateral processes of the palatine (character state 14∶1 of the present study) and the presence of 18 presacral vertebrae (character state 45∶1 of the present study). However, this relationship was not recovered in the present analysis. Both the enlarged posterolateral process of the palatine and the presence of 18 presacral vertebrae appear to be convergently acquired in Podargidae and Cypselomorphae given that *Fluvioviridavis* and *Steatornis* lack an enlarged posterolateral process of the palatine and have 19 presacral vertebrae. Interestingly, when extinct Podargiformes are excluded from the data matrix, analysis of the morphological data matrix results in *Prefica*, *Steatornis* and Podargidae being recovered as successive sister taxa to Cypselomorphae (TL = 167, CI = 0.623, RI = 0.670). This result demonstrates the importance of sampling both stem and crown taxa in phylogeny estimation.

## Discussion

### The status of Fluvioviridavidae

Mayr [4] coined Fluvioviridavidae to encompass two monotypic taxa, *Fluvioviridavis* and *Eurofluvioviridavis*. Additional privately held specimens from the London Clay of Walton-on-the-Naze were also suggested to represent members of this clade [4]. Therefore, we tested the monophyly of Fluvioviridavidae in our phylogenetic analysis by scoring *Fluvioviridavis* and *Eurofluvioviridavis* independently. In no iteration of the analysis (i.e., with different outgroups and with inclusion/exclusion of the molecular and soft-tissue data) was *Eurofluvioviridavis* recovered as the sister-taxon of *Fluvioviridavis*. Furthermore, *Eurofluvioviridavis* was never found within the Podargiformes + Steatornithiformes clade.

A close relationship of *Fluvioviridavis* and *Eurofluvioviridavis* can be rejected based on a detailed comparison of the new specimen of *Fluvioviridavis* and *Eurofluvioviridavis.* Only the features of the dorsal surface of the skulls of *Fluvioviridavis* and *Eurofluvioviridavis* could be directly compared by Mayr and Daniels [3] and Mayr [4]. The new specimen of *Fluvioviridavis* bears a well-preserved palate and parts of both the dorsal and ventral side of the mandible. The palate and mandible of *Fluvioviridavis* is highly divergent from that of *Eurofluvioviridavis*. Whereas the palate of *Fluvioviridavis* involves a fused premaxilla-maxilla-palatine complex, the palatines and maxillae are unfused and well separated in *Eurofluvioviridavis*. Likewise, the mandibles of *Fluvioviridavis* and *Eurofluvioviridavis* differ markedly. The mandible of *Fluvioviridavis* is dorsoventrally flattened, concave dorsally and convex ventrally, and lacks the broad fossae on the lateral side of the mandible as in *Eurofluvioviridavis*. Furthermore, *Eurofluvioviridavis* lacks a midline plantar crest on the tarsometatarsus whereas this crest is well-developed in the new specimen of *Fluvioviridavis* described here. These characters of the palate, mandible, and foot are all synapomorphies uniting *Fluvioviridavis, Masillapodargus,* and Podargidae.

A distant relationship of *Fluvioviridavis* and *Eurofluvioviridavis* has important implications for previous interpretations of the hypothesized relationships of Fluvioviridavidae which were assessed based on scoring both *Fluvioviridavis* and *Eurofluvioviridavis* into a composite terminal [4] and therefore cannot be substantiated. Furthermore, the previous diagnosis of Fluvioviridavidae can no longer be used to confidently assign fragmentary specimens from the London Clay to Fluvioviridavidae because the characters used for the original assignment (both a cup-like scapular articulation of the coracoid and a supracoracoid nerve foramen) occur widely and show complex distributions in distantly related subclades of Aves (e.g., stem Panpsittaciformes, stem Coliiformes).

At present, Fluvioviridavidae includes only *Fluvioviridavis platyrhamphus*. The relationships of *Eurofluvioviridavis* are beyond the scope of this paper, but some osteological evidence such as the semi- or facultatively zygodactyl foot suggests that *Eurofluvioviridavis* may have affinities with some other “higher land bird” clade within the Coronaves rather than with Strisores.

### Evolution of Podargiformes

**Figure 8:**
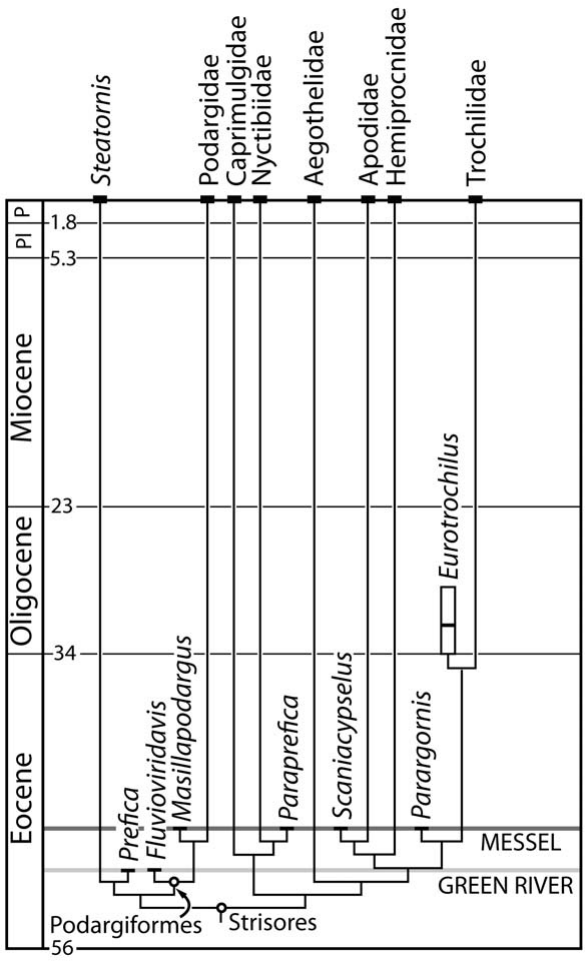
Time calibrated phylogeny of Strisores showing the early diversification by the middle Eocene. The relationships of the Apodiformes were based on Mayr [5], [71]. Time scale based on Gradstein et al. [72] and the ages of Messel and Fossil Butte Member were based on Mertz and Renne [70] and Smith et al. [32], respectively.

Podargiformes have a deep history extending to the Eocene, as first recognized by Mourer-Chauviré [10] and Mayr [8] and elaborated on here. Although fossil members of the group are exceedingly rare, recognition of *Fluvioviridavis* as a stem frogmouth extends the record into the early Eocene. A major stratigraphic gap spanning approximately 37.2–40.4 million years separates the youngest fossil frogmouths from their extant relatives (Fig. 8). The youngest reported records are several partial tibiotarsi and tarsometatarsi assigned to *Quercypodargus olsoni* collected from a late Eocene (MP16: ∼37.2–40.4Ma) horizon in the Quercy fissure fills [10]. These specimens appear to represent stem lineage frogmouths based on the limited morphological information available, though the tibiotarsus is unlike that of any other fossil or living species of Podargiformes in that it has a wide intercondylar incisure [8]. We scored *Quercypodargus* and recovered this taxon within a polytomy with all other Podargiformes (TL = 175, CI = 0.600, RI = 0.700). The relationships of all other taxa in the matrix were the same as in the combined analysis. Because it is so poorly known, *Quercypodargus* could only be scored for four characters in our matrix and its placement among podargiforms was supported by just one character state: tendons of m. flexor digitorum longus and m. flexor hallucis longus enclosed in bony canals (character 87). However, this character could not be scored in the other extinct Podargiformes and is also present in many unrelated avian clades. We concur with Mayr [8] that determining the affinities of *Quercypodargus* will require additional, associated fossil material. The only other records of Podargidae are from Pleistocene cave deposits of Australia. All of these fossils were attributed to the extant species *Podargus strigoides* (e.g., [62])

The morphological divergent Podargiformes are unique among Aves in several aspects of their skeleton, especially their skull. Podargidae have a highly ossified palate, a dorsoventrally flattened and laterally extensive beak with dorsally oriented nares, a rounded mandible with a distinct quadrate-articular locking mechanism, short legs with long tarsometatarsi, a anteroposteriorly short sternum, and relatively elongated, narrow coracoids. The fossil record of Podargiformes indicates that many of the unique cranial characteristics exhibited by crown clade Podargidae (listed above) may have arisen deep within the stem frogmouth lineage. These stem taxa simultaneously lacked most of the postcranial synapomorphies of Podargidae. For example, both *Fluvioviridavis* and *Masillapodargus* have long legs and short tarsometatarsi, anteroposteriorly elongated sterna, and wide coracoids. The morphology of the beak (the namesake of the clade) evolved early in the evolutionary history of the group and was retained for 50 million years; however, the postcranial morphology changed dramatically from the more basal forms to the crown. The retention of plesiomorphic postcranial features such as the wide coracoid and proportionally shorter tarsometatarsus in *Masillapodargus*, a taxon with an essentially a *Podargus*-like skull, further supports this proposed pattern. The conservation of the beak shape may reflect diet and foraging behaviors in Eocene Podargiformes similar to those of extant species.

The diet and prey-capture strategy of extant Podargidae remain incompletely understood because of the nocturnal habits of all living frogmouths. However, members of Podargidae have been recorded consuming a variety of large insects (e.g., grasshoppers and beetles), as well as small vertebrates including mammals, lizards, frogs, and even birds [6]. *Podargus* has been reported to aggressively shake or “beat” their prey with their beaks, before swallowing – a strategy likely facilitated by the strong interlocking quadrate-mandible articulation, solid palate, and the robust mandibular symphysis.

Nearly all Podargidae inhabit primary forests in the Australasia, Oceania, and throughout southeastern Asia regions with the exception of the drier woodland inhabitant, *Podargus strigoides* [6]. Furthermore, the limited data demonstrate that Podargidae are among the most sedentary birds within Aves: none are migratory, there are no seasonal movements, and each solitary pair holds small territories [6]. The paleoenvironment of the two stem frogmouths, *Fluvioviridavis* and *Masillapodargus*, indicate that they lived in widespread sub- or paratropical forests present in the Eocene of North America and Europe ([63], [64]; see refs in [65]). Thus these birds may well have attained a wide Northern Hemisphere distribution during the Paleogene, though they do not appear to have been particularly abundant, at least near the lacustrine settings that are well sampled for Paleogene fossil birds. The presence of podargiforms in North American and Europe during the Eocene is followed by an apparent severe range retraction to their extant distribution. Such a pattern is found in a variety of avian clades represent in the Green River and Messel Formations [5], [40], [53], [66], [67]. A face-value interpretation of the fossil record would suggest a progressive paleobiogeographic range retraction in Podargiformes first from North America and subsequently from Europe, resulting in their present distribution in Australasia, southeastern Asia and Oceana. This pattern, however, may be an artifact of very sparse sampling. Indeed, the ranges of the three known fossil species do not even provide certain evidence that the North American and European records overlap and few fossils have been reported from Australia (e.g., [62]). We likewise have almost no temporal resolution for when major range shifts may have occurred, other than that the disappearance of the clade from Europe must fall within the 37.2–40.4 Ma gap between the youngest stem fossil and the present.

### Early radiation of Strisores

The identification of Podargiformes from the middle Eocene of the North America and Europe demonstrates the antiquity of frogmouths as one of many diverse subclades within Strisores already present by the middle Eocene. Nearly complete skeletons of fossil stem taxa closely related to the extant Steatornithidae (*Prefica nivea,* [2], [8], [55]), Nyctibiidae (*Paraprefica kelleri*, [8], [55], [57]), Apodidae (*Scaniacypselus szarskii*, [68], [69]) and Trochilidae (*Parargornis messelensis,* [15], [57]) are also recognized from early and middle Eocene deposits (Fig. 8). These data indicate that Strisores had undergone both a substantial phylogenetic diversification and major ecological radiation by the time fossiliferous layers of the Messel Formation were deposited (∼47 mya; [70]).

The deep diversification of Strisores demonstrates the importance of fossils to fully understanding the evolutionary history of the clade. Eocene stem representatives of many avian clades are inferred to have had distinct ecologies from their crown clade relatives. Examples include frugivorous stem members of the roller lineage (all extant members of which are predatory) and non-nectivorous stem hummingbirds (reviewed in [5]). Basal Podargiformes, however, appear to have been similarly adapted for hunting relatively large prey compared to extant frogmouths based on a suite of derived cranial features. Additionally, because extant members of Podargidae, Steatornithidae, Caprimulgidae, Nyctibiidae, and Aegothelidae are all nocturnal or crepuscular, it is plausible to infer that the common ancestor of Strisores (and by extrapolation fossil taxa of Podargiformes) was at least crepuscular in habit (see [5], [14], [15] for a detailed discussion). Thus, frogmouths may well have occupied a similar ecological role for over 50 million years.

## Supporting Information

Information S1 **Phylogenetic character list, data matrix, specimens list (molecular and morphological), and references.**
(DOC)
