# Supplementary material for: Podargiform Affinities of the Enigmatic Fluvioviridavis platyrhamphus and the Early Diversification of Strisores (“Caprimulgiformes” + Apodiformes)
Source: PLoS One. 2011 Nov 30;6(11):e26350. doi: 10.1371/journal.pone.0026350 (PMC3227577; doi:10.1371/journal.pone.0026350)
Supplement: Information S1 — Phylogenetic character list, data matrix, specimens list (molecular and morphological), and references. (DOC) [file pone.0026350.s001.doc]

**Supplemental Information**

**Character descriptions and scores. Abbreviations:** (reference: character number)

1. Ossified nasal septum: absent (0); present and largely/completely ossified (1). ([1]: 1)

2. External naris, anterior portion, rim surrounding naris: restricted to the external naris (0); extends anterolaterally onto the dorsal surface of the beak (1). (new)

3. Beak, form: narrow, dorsoventrally tall (0); dorsoventrally flattened (1). (new)

4. Beak, length and form: longer than rest of skull (0); shorter than rest of skull and very wide at its base, with narial openings large and reaching far into its tip (1); shorter than rest of skull and narrow at base (2). ([1]: 2)

5. Rostrum, dense neurovascular pitting: absent (0); present (1). (new)

6. Maxilla, posterior termination: continuous with the jugal bar (0); extends laterally and posteriorly to the contact jugal bar (=angulus tomialis) (1). (modified from [2]: 408)

7. Skull, distinct nasofrontal hinge, i.e., posterior part of beak markedly set off from anterior part of cranium by a furrow: absent (0); present (1). ([3]: 5)

8. Lacrimal, descending process: present and approaches jugal bar (0); absent (1). (modified from [1]: 3)

9. "Horns" projecting posteriorly at front of orbit: absent (0); present (1). (modified from [2]: 383)

10. Sclerotic ossicles: small, average size (0); greatly enlarged (1). (new)

11. Ectethmoid: no anterior or lateral expansion (0); greatly expanded anteriorly, plate-like, with dorsal margin largely fused with frontals (1); greatly expanded anterolaterally, inflated, with dorsal margin largely fused with frontals (2). (modified from [1]: 4)

12. Vomer: not as follows (0); with truncate anterior and bifurcate posterior end (typical of the "aegithognathous" palate) (1). ([1]: 5)

13. Palatine, lateral portion: unexpanded or poorly expanded (0); extremely anterolaterally expanded (1). ([1]: 8)

14. Palatine, strongly protruding posterolaterally directed processes (Mayr 2002: figure 3): absent (0); present (1). ([1]: 7)

15. Palate, internal choana, framed by ventrally directed lamina: absent (0); present (1). (modified from [2]: 442)

16. Internal choana, opening: ventral (0); posterior (1). (new)

17. Palatines: remain separate anterior to the internal choana (0); fused anterior to the internal choana (1). (new)

18. Palatine, anterior process long and slender (Mayr 2002: figure 3): no (0); yes (1). ([1]: 6)

19. Palatine and pterygoid: separate elements (0); fused (1). ([1]: 9)

20. Postorbital process: short (0); elongated, touching (or nearly touching) the jugal bar (1). (modified from [4]: 13)

21. Palatine, lateral portion: flat (0); bears a fossa on the ventral surface that is anterior to the internal choana and is separated from its antimere from a midline lamina (1). (modified from [2]: 455)

22. Pterygoid-basipterygoid contact: present at the posterior portion of or along the midshaft of the pterygoid (0); present and restricted to the anterior portion of the pterygoid (1); absent (2). (modified from [1]: 10)

23. Paroccipital processes: projected laterally and slightly ventrally (0); strongly ventrally protruding, pointed, and at or ventral to the level of the articular surface of the quadratomandibular joint (1). ([1]: 11)

24. Cone-like bony protrusion at posterior margin of the optic nerve foramen: absent (0); present (1). ([1]: 12)

25. Jugal bar and mandible rami, lateral view: straight or nearly straight (0); strongly bowed, so that lateral margin of skull is convex (1). ([4]: 18)

26. Parietal area, temporal fossae: absent or far from the midline (0); meeting or almost meeting at midline of skull (1). (modified from [4]: 19)

27. Quadrate, orbital process: anteriorly elongated (0); greatly reduced, short process (1); absent (2). (modified from [1]) ORDERED

28. Quadrate, proximal portion, lateral condyle separated from the medial condyle by a deep but narrow furrow: no (0); yes (1). ([1]: 14)

29. Quadrate, otic process, dorsal margin of posterior surface: smooth (0); with many small pneumatic foramina (1). (modified from [1]: 15)

30. Mandible, distal part of mandibular rami very narrow, thus the mandibular symphysis is very short (less than a third of maximum width of rami): no (0); yes (1). ([1]: 16)

31. Articulation between the quadrate and the mandible, incorporation of a locking mechanism by means of a laterally directed peg on the quadrate fitting into a medially open concavity of the articular portion of the mandible: absent (0); present (1). ([2]: 710)

32. Mandible, posterior edge of the articular portion, in dorsal view: mediolaterally flat (0); rounded posteriorly (1). (new)

33. Mandibular rami, ventral margin, lateral view: distinctly decurved (0); virtually straight (1); present, strongly recurved (2). (modified from [2]: 673)

34. Mandible, anterior portion of the symphysis: distinctly pointed (0); nearly rounded (1). (new)

35. Mandibular rami, pronounced, monotonic curvature producing continuous lateral concavity: absent (0); present (1). ([2]: 658)

36. Mandible with intraramal joint and posterior half of mandibular rami greatly widened (mediolateral width greater than dorsoventral extension) and dorsoventrally flattened: no (0); yes (1). ([1]: 17)

37. Mandible, proximal end small, with stout medial process and medial cotyla forming a narrow sulcus: no (0); yes (1). ([1]: 18)

38. Atlas, opening for odontoid process: dorsally open incisura fossa (0); enclosed foramen (1). ([1]: 19)

39. Axis, transverse foramina: present (0); absent (1). ([1]: 20)

40. Third cervical vertebra, osseous bridge from transverse process to the postzygapophysis: absent (0); present (1). ([1]: 21)

41. Fifth cervical vertebra, osseous bridge from costal process to midsection of the centrum: no (0); yes (1). ([1]: 22)

42. Thoracic vertebrae 1 and 2: ventral process absent or present and blade-like (0); "trifid" processus ventralis with 2 lateral projections on thoracic 1 and 2 (1). (new)

43. Thoracic vertebrae 1 and 2, ventral tip of ventral process forming large horizontal plate: no (0); yes (1). ([1]: 23)

44. Pygostyle, body perforated at posteroventral end ([3]: figure 6G): yes (0); no (1). ([4]: 30)

45. Number of presacral vertebrae (all vertebrae anterior to synsacrum): 19 or more (0); 18 (1); 17 (2). ([1]: 24)

46. Furcula, omal tip with distinct, laterally protruding acrocoracoid articular facet: no (0); yes (1). ([1]: 25)

47. Coracoid, articular surface for the scapula: cup-like (0); flat (1). ([1]: 26)

48. Coracoid, supracoracoid nerve foramen: present (0); absent (1). ([1]: 27)

49. Coracoid, lateral process: short (0); long (1). ([1]: 28)

50. Coracoid, small foramina ventral to acrocoracoid process: absent (0); present (1). (new)

51. Scapula, blade: generally curved (0); strait and distal tip is deflected (1). (new)

52. Sternum, spina interna rostri: absent (0); present (1). ([4]: 39)

53. Sternum, articular surface for the coracoid weakly saddle-shaped or convex: no (0); yes (1). ([1]: 31)

54. Sternum, spina externa forming a ridge that extends dorsoventrally over entire sternum rostrum: no (0); yes (1). ([1]: 29)

55. Sternum, number of notches/fenestrae: four notches/fenestrae (0); two notches/fenestrae (1); without notches/fenestrae (2). (modified from [1]: 32)

56. Sternum, perforations: posteriorly open (0); posteriorly closed (1). (new)

57. Sternum: lateral and intermediate trabeculae short and subequal (0); very long lateral trabeculae and short trabecula intermedia (1). (new)

58. Humerus, proximal end, posterior prominence of ventral tubercle distinctly greater than that of head of the humerus: no (0); yes (1). ([1]: 34)

59. Humerus, proximal end, deltopectoral crest: proximodistally elongated (0); proximodistally narrow, strongly protruding and tapering (1). (modified from [4])

60. Humerus, proximal portion, distal end of bicipital surface: indistinct and continuous with the shaft (0); distinct and extends ventral to the shaft (1). (new)

61. Humerus, proximal end, transverse sulcus very deep, long and rectangular-shaped: no (0); yes (1). ([1]: 33)

62. Humerus, distal end, fossa for the m. brachialis deep and sharply delimited: no (0); yes (1). ([1]: 36)

63. Humerus, greatly abbreviated and stocky (ratio of length of bone: width of shaft in midsection less than 7.0): no (0); yes (1). ([1]: 37)

64. Humerus: tibiotarsus proportions: tibiotarsus significantly longer (0); approximately equal in length (1); humerus significantly longer (2). (new) ORDERED

65. Humerus, distal end, ventral supracondylar tubercle: low tubercle (0); elongated and narrow process (1). ([1]: 35)

66. Ulna distinctly exceeding humerus in length: no (0); yes (1). ([1]: 39)

67. Radius, distal end with marked tubercle on ventral side of shaft, opposite to carpal tubercle of ulna: no (0); yes (1). ([1]: 38)

68. Carpometacarpus, intermetacarpal process: absent or small (0); well-developed, reaching metacarpal III (1); absent but tendon of musculus extensor carpi ulnaris inserts on metacarpal III as it does in taxa with a intermetacarpal process (2). ([4]: 50)

69. Carpometacarpus, projection of distal end of metacarpal III: shorter than or subequal to metacarpal II (0); longer than metacarpal II (1). ([1]: 40)

70. Ulnare, length of crus longum versus crus breve length: crus breve longer (0); crus longum longer (1). ([1]: 41)

71. Manual phalanx II-1, cranial face: narrow (0); dorsoventrally widened, giving phalanx a T-shaped cross section (1). (new)

72. Manual phalanx I-1 (=alulae), claw: present (0); absent or rudimentary in adulthood (1). ([1]: 42)

73. Manual phalanx II-1, well-developed internus indicis process: absent (0); present (1). ([1]: 44)

74. Manual phalanx II-1, dorsal fossa: single depression (0); divided into two depressions or fenestrae separated by a distinct oblique bulge (1). ([1]: 43)

75. Manual phalanx II-2, distal end: straight or slight posterior expansion (0); posterior expansion well developed (1). (new)

76. Pelvis, ilioischiadic foramen: open (0); closed (1). ([1]: 45)

77. Pelvis, posterior portion, juncture of the ilium and the ischium, posteriorly directed point: present (0); absent (1). (new)

78. Pelvis wide in mediolateral direction, width across antitrochanters as much or more than length of synsacrum: no (0); yes (1). ([1]: 46)

79. Pelvis, well-developed preacetabular tubercle: present (0); absent (1). ([1]: 47)

80. Pelvis, terminal portion of the ischium slender, touching pubis at an angle of 45^o^ - 90^o^, thus creating a very wide ischiopubic fenestra: no (0); yes (1). ([4]: 63)

81. Tibiotarsus, length: distinctly longer than carpometacarpus (0); shorter or about equal length to the carpometacarpus (1). ([4]: 65)

82. Tibiotarsus, distal end, supratendinal bridge: ossified (0); unossified (1). ([1]: 48)

83. Tibiotarsus, distal end, tibial cartilage trochlea sulcus: shallow (0); deep (1). ([1]: 49)

84. Intertarsal sesamoid: absent (0); present (1). ([1]: 50)

85. Tarsometatarsus, length: more than half as long as carpometacarpus (0); less than half as long as carpometacarpus (1). (modified from [1]: 51)

86. Tarsometatarsus, hypotarsus passing into a well developed midline plantar crest (=crista medianoplantaris); medial parahypotarsal fossa very marked and proximal part of the medial margin forming a sharp ridge: no (0); yes (1). ([4]: 71)

87. Tarsometatarsus, hypotarsus, tendons of musculus flexor digitorum longus and musculus flexor hallucis longus enclosed in bony canals: no (0); yes (1). ([1]: 53)

88. Tarsometatarsus, arcus extensorius (ossified retinaculum extensorium): absent (0); present (1). ([1]: 54)

89. Tarsometatarsus, canalis interosseus distalis: present (0); absent (1). ([1]: 55)

90. Tarsometatarsus, midline plantar crest (=crista medianoplantaris): absent (0); present (1). (new)

91. Tarsometatarsus, tubercle for m. tibialis cranialis very prominent: no (0); yes (1). ([1]: 52)

92. Hallux, proximal phalanx with proximal end greatly widened: no (0); yes (1). ([4]: 78)

93. Third and fourth toe coalescent at least over length of basal phalanx of third toe: no (0); yes (1). ([4]: 79)

94. Second and third phalanx of fourth toe greatly abbreviated, measuring less than half the length of the fourth phalanx: no (0); yes (1). ([1]: 56)

95. Cerebellum with reduced anterior lobe, particularly small folia II and III, and relatively large posterior lobe: no (0); yes (1). ([1]: 69)

96. Caeca: absent (0); present (1). ([1]: 68)

97. Rictal bristles: absent (0); present (1). ([1]: 67)

98. Villi at the bases of the basal-most downy barbules of breast feathers: absent (0); present (1). ([4]: 98)

99. Wing: diastataxic (0); eutaxic (1). ([1]: 66)

Soft tissue characters not included in the combined analysis (see discussion) because they are scored by the family level by [1].

100. M. splenius capitis with cruciform origin: no (0); yes (1). [1]: 57)

101. M. ambiens: present (0); absent (1). ([1]: 58)

102. M. iliofemoralis externus: present (0); absent (1). ([1]: 59)

103. M. flexor cruris lateralis, pars accessoria: present (0); absent (1). ([1]: 60)

104. M. caudofemoralis, pars pelvica: present (0); absent (1). ([1]: 61)

105. M. fibularis longus: present (0); absent (1). ([1]: 62)

106. M. popliteus: present (0); absent (1). ([1]: 63)

107. Vinculum between tendons of musculus flexor perforans et perforatus digiti III and musculus perforatus digiti III: present (0); absent (1). ([1]: 64)

Character scores. **Abbreviations:** com, combined; *Eurofluvio*, *Eurofluvioviridavis*.

10 20 30 40 50 60

*Crypturellus undulatus* 000000?00000000000100000000010000100000000010011100100100000

*Trogon massena* 100201100000000000000000000010000000011000010011011000000000

*Leptosomus discolor* 100000000?100000000101000000000000000??????1?100110000000100

*Eurypyga helias* 000000000010000000000100010000000000000000010000100000100100

*Steatornis caripensis* 100211?1000000101000000001000?000000000101010001100000100000

*Prefica nivea* ????????????????????????01???0?0?0000???????????1?0?0?000?00

*Nyctibius griseus* 001100010120110000?00011102101000001101111011101110000000100

*Nyctibius grandis* 00110001012011000000001110210100000110111101?101110000000100

*Quercypodargus olsoni* ????????????????????????????????????????????????????????????

*Masillapodargus longipes* ??101??101????1??00??20?0??0?0?111100????1????010?1?0?000000

*Eurofluv. robustipes* ?0100??????????0000??2???????0?0?000???????0???0??0??????00?

FMNH *Fluvioviridavis* 1?101101000?001010000200011000?110100001????0???????????????

SMNK *Fluvioviridavis* ?1101?0100???????????????01??????0?????????1?0001?0???00??00

*Fluvioviridavis* com 11101101000?001010000200011000?110100?01???100001?0???00??00

*Podargus strigoides* 11101111110?010110011200011000111110000111011011101000001000

*Batrachostomus auritus* 11101111110?010110011200011000111110000111011011101000001000

*Caprimulgus carolinensis* 001100000020110001000011102101000001110101001111101000100100

*Paraprefica kelleri* 001100??0???1100??00?0??102??1?000011?????????01??0???000?00

*Aegotheles cristatus* 001100?1000101000100010000111?000000101001011110010000010000

*Hemiprocne comata* 001100?1001101000100010000111?000000001001111100011011210111

*Amazilia tzacatl* 001000?1001100000100010000111?0000000111010111000000110?0100

*Aerodramus vanikorensis* 001100?10?1101000100010000111?000000011001111100010011110111

70 80 90 100 107

*Crypturellus undulatus* 0000000000?10000?000000000000?0000010?100000000

*Trogon massena* 0102010011110001011100000010110010?????????????

*Leptosomus discolor* 01020100001?11010010000?0010?11000?????????????

*Eurypyga helias* 11000100000?01010010000?0000000000?????????????

*Steatornis caripensis* 1102010011111001011011001000011001011?001101100

*Prefica nivea* ?102?100011?01010???1???10?0??1000?????????????

*Nyctibius griseus* 1002010001011111101001011010000000?1???01101011

*Nyctibius grandis* 11020100?10111111010?1011010000000?????01101011

*Quercypodargus olsoni* ?????????????????????00???1??1?????????????????

*Masillapodargus longipes* 0101?1?0011?010?????0???0??????000?????????????

*Eurofluv. robustipes* ??0??1?00?000101???0????1????0?000?????????????

FMNH *Fluvioviridavis* ???????????????10??0??0011???1?000?????????????

SMNK *Fluvioviridavis* ?100?1000??0110100100?0?1??????000?????????????

*Fluvioviridavis* com?100?1000??0110100100?0011???1?000?????????????

*Podargus strigoides* 0101010001110101001000000010110000011?001101001

*Batrachostomus auritus* 01020100111?01010010000?0010110000011?001101011

*Caprimulgus carolinensis* 10020100010111010110000?00001000001110001101001

*Paraprefica kelleri* 100??1?001010?01?01001??10?0???0?0?????????????

*Aegotheles cristatus* 1000010001010101011100000000110100101?011101111

*Hemiprocne comata* 1111111001011111111101101?01101??1?00??111?????

*Amazilia tzacatl* 00110001100110111111001000011110111001111111111

*Aerodramus vanikorensis* 10101110010111110111001000011111011000111111111

**Sources of character scorings**

*Aegotheles* *cristatus* USNM 632141

*Aerodramus* *vanikorensis* USNM 557188

*Amazilia* *tzacatl* USNM 613414

*Batrachostomus* *auritus* USNM 430279

*Caprimulgus* *carolinensis* NCSM 18510

*Crypturellus* *undulatus* AMNH 6480

*Eurofluvioviridavis robustipes* SMF-ME 10783a + 10783b, SMNK.PAL.3835 [5], [6]

*Eurypyga* *helias* USNM 614111

*Hemiprocne* *comata* USNM 488343

*Fluvioviridavis* *platyrhamphus* SMNK.PAL.2368a+b holotype, FMNH PA 607

*Leptosomus* *discolor* USNM 291844

*Masillapodargus* *longipes* SMNK.PAL.1083 holotype, SMF-ME 1415a+b, SMNK.PAL.552a+b, SMNK.PAL.557, SMF-ME 3404a+b, SMF-ME 3405a+b., SMF-ME 3406, [7], [8]

*Nyctibius* *grandis* USNM 615095

*Nyctibius* *griseus* USNM 555945

*Paraprefica kelleri* SMF-ME 3376 holotype, SMF-ME 1635a+b, SMF-ME 1926, SMF-ME 2553, SMF-ME 3377a+b, SMNK.PAL.938, SMF-ME 3578, SMF-ME 3727a+b, SMF-ME 1760 [7], [4]

*Podargus* *strigoides* USNM 612707

*Prefica nivea* USNM 336278

*Steatornis* *caripensis* USNM 560206, 560152

*Trogon* *massena* USNM 612327, 612328

**Molecular data sources for each taxon used in the combined analysis.**

|  | cytochrome b | c-myc ex3 | RAG |
| --- | --- | --- | --- |
| *Aegotheles cristatus* | X95775  (Mariaux and Braun, 1996 [9]) | FJ588483  (Braun and Huddleston 2009 [10]) | - |
| *Aerodramus vanikorensis* | FJ588453  (Braun and Huddleston 2009 [10]) | EU738244  (Hackett et al. 2008 [11]) | - |
| *Amazilia tzacatl handleyi* | FJ588452  (Braun and Huddleston 2009 [10]) | FJ588479  (Braun and Huddleston 2009 [10]) | - |
| *Batrachostomus septimus* | EF100673  (Cleere et al. 2007 [12]) | EU738255  (Hackett et al. 2008 [11]) | DQ482613  (Barrowclough et al. 2006 [13]) |
| *Caprimulgus carolinensis* | FJ588442  (Braun and Huddleston 2009 [10]) | FJ588461  (Braun and Huddleston 2009 [10]) | DQ482627  (Barrowclough et al. 2006 [13]) |
| *Crypturellus undulatus* | AY139629  (Garcia-Moreno et al. 2003 [14]) | - | - |
| *Eurypyga helias* | - | EU738293  (Hackett et al. 2008 [11]) | DQ881806  (Ericson et al. 2006 [15]) |
| *Hemiprocne comata* | FJ588455  (Braun and Huddleston 2009 [10]) | FJ588481  (Braun and Huddleston 2009 [10]) | - |
| *Leptosomus discolor* | AF407449  (Kirchman et al. 2002 [16]) | EU738312  (Hackett et al. 2008 [11]) | AY233361  (Mayr et al 2003 [17]) |
| *Nyctibius grandis* | EU344977  (Pratt et al. 2008 [18]) | EU738328  (Hackett et al. 2009 [11]) | DQ482612  (Barrowclough et al. 2006 [13]) |
| *Podargus strigoides* | EF100672  (Cleere et al. 2007 [12]) | EU738354  (Hackett et al. 2008 [11]) | EF373496  (Pereira et al. 2007 [19]) |
| *Steatornis caripensis* | EF100675  (Cleere et al. 2007 [12]) | FJ588476  (Braun and Huddleston 2009 [10]) | DQ482611  (Barrowclough et al. 2006 [13]) |
| *Trogon massena* | DQ364134  (Weir 2007 [20]) | - | AY625228  (Moyle 2005 [21]) |

**References**

1. Mayr G (2010) Phylogenetic relationships of the paraphyletic 'caprimulgiform' birds (nightjars and allies). Journal of Zoological Systematics and Evolutionary Research 48: 126-137.

2. Livezey BC, Zusi RL (2006) Higher-order phylogeny of modern birds (Theropoda, Aves: Neornithes) based on comparative anatomy. I. Methods and characters. Bulletin of the Carnegie Museum of Natural Histroy 37: 1-544.

3. Mayr G, Clarke JA (2003) The deep divergences of neornithine birds: a phylogenetic analysis of morphological characters. Cladistics 19: 527-553.

4. Mayr G (2005) The Palaeogene Old World potoo *Paraprefica* Mayr, 1999 (Aves, Nyctibiidae): its osteology and affinities to the New World Preficinae Olson, 1987. Journal of Systematic Palaeontology 3: 359-370.

5. Mayr G, Daniel M (2001) A new short-legged landbird from the early Eocene of Wyoming and contemporaneous European sites. Acta Palaeontologica Polonica 46: 393-402.

6. Mayr G (2005) A *Fluvioviridavis*-like bird from the Middle Eocene of Messel, Germany. Canadian Journal of Earth Sciences 42: 2021-2037.

7. Mayr G (1999) Caprimulgiform birds from the Middle Eocene of Messel (Hessen, Germany). Journal of Vertebrate Paleontology 19: 512-532.

8. Mayr G (2001) Comments on the osteology of *Massillapodargus* *longipipes* Mayr 1999 and *Paraprefica* *major* Mayr 1999, caprimulgiform birds from the Middle Eocene of Messel (Hessen, Germany). Neues Jahrbuch für Geologie und Paläontologie, Monatshefte 2001: 65-76.

9. Mariaux J, Braun MJ (1996) A molecular phylogenetic survey of the nightjars and allies (Caprimulgiformes) with special emphasis on the potoos (Nyctibiidae). Molecular Phylogenetics and Evolution 6: 228-244.

10. Braun MJ, Huddleston CJ (2009) A molecular phylogenetic survey of caprimulgiform nightbirds illustrates the utility of non-coding sequences. Molecular Phylogenetics and Evolution 53: 948-960.

11. Hackett SJ, Kimball RT, Reddy S, Bowie RCK, Braun EL, et al. (2008) A phylogenomic study of birds reveals their evolutionary history. Science 320: 1763-1768.

12. Cleere N, Kratter AW, Steadman DW, Braun MJ, Huddleston CJ, et al. (2007) A new genus of frogmouth (Podargidae) from the Solomon Islands - results from a taxonomic review of *Podargus ocellatus inexpectatus* Hartert 1901. Ibis 149: 271-286.

13. Barrowclough GF, Groth JG, Mertz LA (2006) The RAG-1 exon in the avian order Caprimulgiformes: phylogeny, heterozygosity, and base composition. Molecular Phylogenetics and Evolution 41: 238-248.

14. García-Moreno J, Sorenson MD, Mindell DP (2003) Congruent avian phylogenies inferred from mitochondrial and nuclear DNA sequences. Journal of Molecular evolution 57: 27-37.

15. Ericson PGP, Anderson CL, Britton T, Elzanowski A, Johansson US, et al. (2006) Diversification of Neoaves: integration of molecular sequence data and fossils. Biology Letters 4: 543-547.

16. Kirchman JJ, Hackett SJ, Goodman SM, Bates JM (2002) Phylogeny and systematics of ground rollers (Brachypteraciidae) of Madagascar. Auk 118: 849-863.

17. Mayr G, Manegold A, Johansson US (2003) Monophyletic groups within 'higher land birds' - comparison of morphological and molecular data. Journal of Zoological Systematics and Evolutionary Research 41: 223-248.

18. Pratt R, Gibb G, Morgan-Richards M, Phillips M, Hendy M, et al. (2009) Towards resolving deep Neoaves phylogeny: data, signal enhancement and priors. Molecular Biology and Evolution 26: 313-326.

19. Pereira SL, Johnson KP, Clayton DH, Baker AJ (2007) Mitochondrial and nuclear DNA sequences support a Cretaceous origin of Columbiformes and a dispersal-driven radiation in the Paleogene. Systematic Biology 56: 656-672.

20. Weir JT (2007) Divergent timing and patterns of species accumulation in lowland and highland Neotropical birds. Evolution 60: 842-855.

21. Moyle RG (2005) Phylogeny and biogeographical history of Trogoniformes, a pantropical bird order. Biological Journal of the Linnean Society 84: 725-738.
